# Electrical Properties of Cu-Based Coordination Complexes: Insights from In Situ Impedance Spectroscopy

**DOI:** 10.3390/molecules30010082

**Published:** 2024-12-29

**Authors:** Jana Pisk, Marko Dunatov, Martina Stojić, Nenad Judaš, Ivica Đilović, Marta Razum, Luka Pavić

**Affiliations:** 1 Department of Chemistry, Faculty of Science, University of Zagreb, Horvatovac 102a, 10000 Zagreb, Croatia; martina.stojic@chem.pmf.hr (M.S.); judas@chem.pmf.hr (N.J.); idilovic@chem.pmf.hr (I.Đ.); 2 Ruđer Bošković Institute, Bijenička Cesta 54, 10000 Zagreb, Croatia; marko.dunatov@irb.hr (M.D.); marta.razum@irb.hr (M.R.)

**Keywords:** copper complexes, (thio)carbohydrazones, aroylhydrazones, impedance spectroscopy, electrical properties

## Abstract

This study examines the influence of ligand design on the structural, optical, and electrical properties of copper-based coordination complexes. Ligands H_2_L^1^ and H_2_L^2^ were synthesized via the reaction of 5-nitrosalicylaldehyde with 2-hydroxy- or 4-hydroxybenzhydrazide. H_4_L^3^ was obtained from the reaction of carbohydrazide and salicylaldehyde, while H_4_L^4^ was prepared by condensing 4-methoxysalicylaldehyde with thiocarbohydrazide. The research focuses on two key design elements: (1) the effect of hydroxyl group positioning on the aroyl ring in hydrazide ligands (H_2_L^1^ vs. H_2_L^2^) and (2) the impact of carbonyl versus thiocarbonyl groups and aldehyde substituents in hydrazone ligands (H_4_L^3^ vs. H_4_L^4^). The resulting complexes, [Cu_2_(L^1^)_2_], [Cu_2_(L^2^)_2_(MeOH)_3_], [Cu_2_(L^3^)(H_2_O)_2_], and [Cu_2_(L^4^)(H_2_O)_2_], were synthesized and characterized using attenuated total reflectance infrared (IR-ATR) spectroscopy, thermogravimetric analysis (TG), and UV-Vis diffuse reflectance spectroscopy. Their electrical properties were investigated using solid-state impedance spectroscopy (IS). The crystal and molecular structure of the complex [Cu_2_(L^2^)_2_(MeOH)_3_]∙MeOH was determined by single-crystal X-ray diffraction (SCXRD). This study underscores the pivotal role of ligand modifications in modulating the functional properties of coordination complexes, offering valuable insights for the advancement of materials chemistry.

## 1. Introduction

Schiff bases and their metal complexes have garnered significant scientific interest due to their pivotal roles in the main group and transition metal coordination chemistry, a consequence of their straightforward synthesis and structural diversity [1,2]. These compounds are typically synthesized through a condensation reaction between primary amines and active carbonyl compounds in the presence of an appropriate solvent, preferably alcohol [3]. The corresponding metal complexes are prepared by reacting Schiff base ligands with metal precursors in controlled stoichiometric ratios under suitable experimental conditions [4,5]. The versatility of Schiff bases and their metal complexes is reflected in their wide-ranging applications, which include their use as chelating ligands in coordination chemistry, catalysts, dyes, polymerization initiators, and luminescent materials [6,7,8,9,10,11,12].

**Scheme 1:**
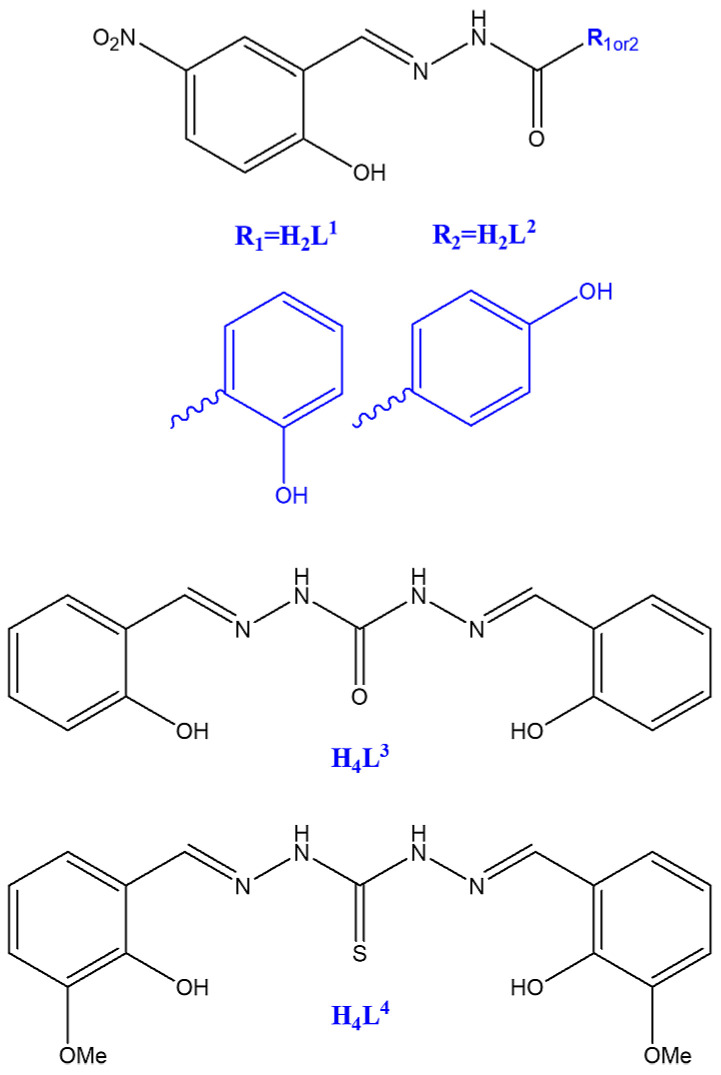
Ligands used in the presented research.

Furthermore, Schiff bases and their metal complexes exhibit notable biological activities, having been investigated as antibacterial, antifungal, antitumor, and antiviral agents, as well as insecticides. The advent of clinically significant metal-based drugs such as cisplatin in the 1970s and auranofin in the 1990s has further driven the exploration of metal complexes as potential therapeutic agents. Among these, copper-based complexes have shown promise as anticancer candidates, with studies revealing their ability to induce cancer cell death via diverse mechanisms, including proteasome inhibition, generation of reactive oxygen species (ROS), and DNA damage [13,14]. These findings underscore the multifaceted nature of Schiff bases and their metal complexes in both fundamental and applied research domains. Another significant application of copper-based complexes lies in their use as semiconductors. The incorporation of transition metals into the organic ligands can enhance the electrical properties of these materials, making them suitable for various electronic and optoelectronic applications. The ability of copper to adopt multiple oxidation states, coupled with the tuneable electronic structure of Schiff base ligands, provides a platform for designing materials with improved conductivity and tailored energy band gaps. This synergy between organic ligands and metal centres offers promising pathways for advancements in semiconductor technology, including organic field-effect transistors (OFETs), photovoltaic cells, and light-emitting diodes (LEDs) [15,16,17]. Structural parameters, including ligand environment, solvent coordination, and metal centres typically influence the conduction mechanisms within such complexes. In this regard, we have reported the electrical properties of molybdenum- and vanadium-based complexes with a similar class of Schiff base ligands [18,19,20,21]. This study aimed to expand the scope of research into copper-based materials by exploring the effects of ligand design on their properties. The selected ligands, shown in Scheme 1, were chosen to investigate two key factors: (1) the impact of hydroxyl position on the aroyl ring of hydrazide (H_2_L^1^ vs. H_2_L^2^) and (2) the influence of carbonyl versus thiocarbonyl groups in hydrazide ligands, along with variations in substituents on the aldehyde moiety of hydrazone ligands (H_4_L^3^ vs. H_4_L^4^). By systematically varying these structural elements, the study aimed to elucidate how these modifications affect the coordination environment, electronic properties, and functional performance of the resulting copper complexes, providing deeper insights into their structure–property relationships.

## 2. Results and Discussion

### 2.1. Preparation and Spectroscopic and Thermal Characterization

The preparation of all the ligands used in this research has been previously reported [18,22,23,24,25]. The freshly prepared ligands were analyzed using IR spectroscopy to confirm their structural features. The IR spectra for ligands H_2_L^1^ and H_2_L^2^ exhibits similar characteristics. Notable absorption bands include the C=O stretching at 1651 cm^−1^ for both ligands. Bands at 1601 cm^−1^ and 1588 cm^−1^ for H_2_L^1^ and H_2_L^2^, respectively, are attributed to C=N stretching. Additionally, C–O_phenolic_ vibrations are observed at 1275 cm^−1^ for H_2_L^1^ and 1238 cm^−1^ for H_2_L^2^.

For ligands H_4_L^3^ and H_4_L^4^, characteristic bands appear at 3241 cm^−1^ and 3238 cm^−1^ (N–H), 1616 cm^−1^ and 1610 cm^−1^ (C=N), 1533 cm^−1^ (C–N), and 744 cm^−1^ (C=S). The DSC analysis provided insights into the melting points and purity of the ligands. For H_2_L^1^ and H_2_L^2^, previously reported DSC results [18] were confirmed by the freshly prepared samples, showing identical thermal behaviour. The endothermic peak for H_4_L^3^ appeared at 197 °C, indicating its melting point, while H_4_L^4^ displayed a melting onset at 213 °C.

The copper complexes were synthesized by reacting [Cu(OAc)_2_]·H_2_O with the respective ligands in methanol, yielding dark green powders. The complex derived from H_2_L^1^ displayed a weak Cu–O stretching band at 664 cm^−1^, with additional bands at 1576 cm^−1^ and 1236 cm^−1^ corresponding to C=N_imine_ and C–O_phenol_, respectively. Similarly, the complex formed with H_2_L^2^ exhibited bands at 640 cm^−1^ (Cu–O), 1602 cm^−1^ (C=N_imine_), and 1271 cm^−1^ (C–O_phenol_). The observed absorption bands are consistent with those reported in the literature for similar classes of copper complexes [26,27].

**Figure 1:**
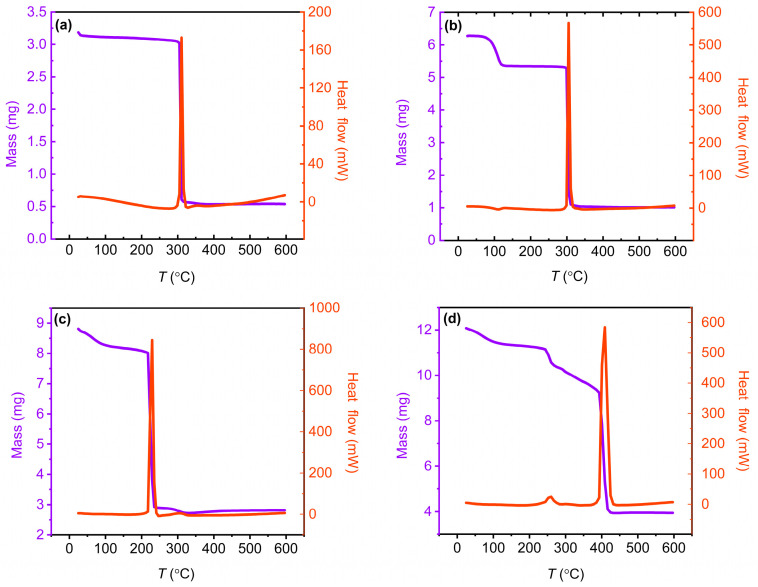
The TG/DSC curves for the complexes: (**a**) [Cu_2_(L^1^)_2_], (**b**) [Cu_2_(L^2^)_2_(MeOH)_3_], (**c**) [Cu_2_(L^3^)(H_2_O)_2_], and (**d**) [Cu_2_(L^4^)(H_2_O)_2_]. Violet cure presents mass loss, while orange one is heat flow.

**Scheme 2:**
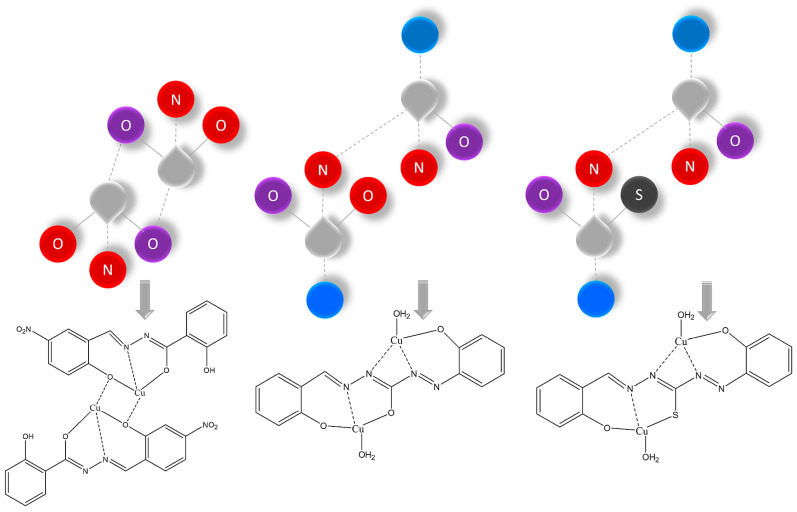
(**above**) Abstract presentation of supposed Cu structures. Grey teardrop presents Cu atom, and purple O is the aldehyde part of the ligand, while red O, N, and S are from the hydrazide part of the ligand. The blue sphere presents a water molecule. (**below**) Supposed structural and molecular formulas.

Thermogravimetric analysis (TG) of the complex obtained from H_2_L_1_ revealed a single-step decomposition in the range of 307–345 °C, indicating a stable thermal profile (Figure 1a,b). In contrast, the complex derived from H_2_L^2^ showed a two-step decomposition process, with an initial mass loss between 92 and 118 °C, likely corresponding to solvent removal, followed by a second decomposition phase between 324 and 344 °C. An interesting property observed from the TG/DSC curves is the decomposition of the Cu complex in a very narrow temperature range (around 30 °C), being unusual for the coordination complex decomposition. The final residue was analyzed as CuO due to its colour and the overlapping of the residue’s IR spectra and the IR spectra of the commercially available CuO. Based on the IR and TG data, the synthesized complexes were identified as dinuclear copper complexes, analogous to previously published compounds. Specifically, the reaction between copper(II) acetate and ligands derived from salicylaldehyde and 4-hydroxybenzhydrazide is known to yield dinuclear complexes with the general formula [Cu_2_(L)_2_], [26]. In this study, the prepared complexes were assigned the structures [Cu_2_(L^1^)_2_] and [Cu_2_(L^2^)_2_(MeOH)_3_]; see Scheme 2. The presence of methanol in the latter was additionally confirmed via IR-ATR spectroscopy, which showed a distinct band at 1025 cm^−1^ corresponding to MeOH. These findings provide a characterization of the ligands and their resulting copper complexes, aligning with established literature precedents [26].

Next, two Cu complexes were obtained through the synthesis of [Cu(OAc)_2_]·H_2_O and H_4_L^3or4^. The IR spectra indicated the absence of the band characteristic for C=O and C=S and the existence of bands at 1600 and 1240 cm^−1^ attributed to C=N_imine_ and C–O_phenol_, respectively. The TG of both complexes, shown in Figure 1c,d, indicated an almost immediate mass loss for the prepared complex obtained from the ligand H_4_L^3^ in the temperature range 50–135 °C and for the complex obtained from the ligand H_4_L^4^ in the range 50–120 °C. Further heating caused complex decomposition, at 237–328 and 253–442 °C, respectively. Based on the results of TG and IR data, it was assumed that both complexes have the analogue formula [Cu_2_(L^3or4^)(H_2_O)_2_]. This is supported by the similar findings published in the literature [28,29,30]; see Scheme 2. When the TG results of the dinuclear copper complexes, [Cu_2_(L^1^)_2_] and [Cu_2_(L^2^)_2_(MeOH)_3_], were compared, a notable difference emerges between the complexes derived from the ligands H_4_L^3^ and H_4_L^4^. The complex is obtained from ligand H_4_L^3^, which features a bridging ONO-NNO donor, and exhibits decomposition within a relatively narrow temperature range. In contrast, the complex featuring ligand H_4_L^4^, characterized by its ONS-NNO donor configuration, undergoes decomposition across a significantly broader temperature range. This disparity in thermal stability suggests a difference in the robustness of the ligand–chelate interactions and the overall structural integrity of the complexes, potentially influencing their reactivity and applications in various chemical contexts.

After multiple experimental trials, the high-quality monocrystals of the Cu complex were successfully obtained by the recrystallization of complex [Cu_2_(L^2^)_2_(MeOH)_3_] from methanol.

### 2.2. Description of Molecular and Crystal Structure for Cu Dimer

**Figure 2:**
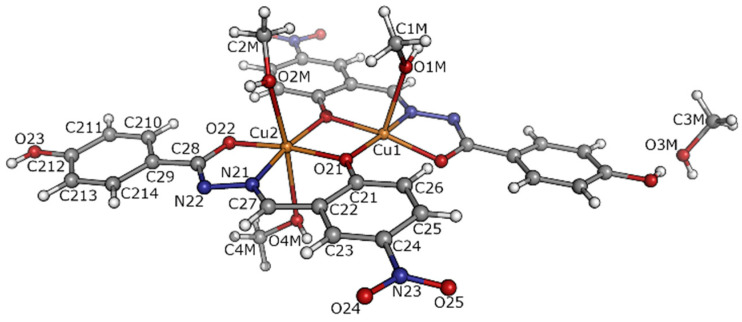
The view of the asymmetric unit of [Cu_2_(L^2^)_2_(MeOH)_3_]∙MeOH with the atom-labelling scheme. For clarity, the labelling of only one ligand is shown. The other molecule is essentially identical in appearance and has the same atom-numbering scheme but starting with 1 (e.g., atom C113 in molecule ligand 1 is labelled C213 in ligand molecule 2).

The crystal and molecular structure of the complex [Cu_2_(L^2^)_2_(MeOH)_3_]∙MeOH was determined by single-crystal X-ray diffraction (Table 1). A dinuclear copper(II) centre features two Cu(II) ions, two doubly deprotonated ligand molecules, and three methanol molecules within its asymmetric unit (Figure 2 depicts the atom-labelling scheme). The Cu1 ion is pentacoordinated, while Cu2 is hexacoordinated. Both copper centres are coordinated by two nearly planar tridentate ONO donor ligands. The coordination spheres are completed by three methanol molecules. The geometry around Cu1 is best described as distorted square pyramidal, whereas Cu2 adopts a distorted elongated octahedral coordination. Relevant bond distances, detailed in Table 2, are consistent with those reported for analogous copper complexes [26].

**Figure 3:**
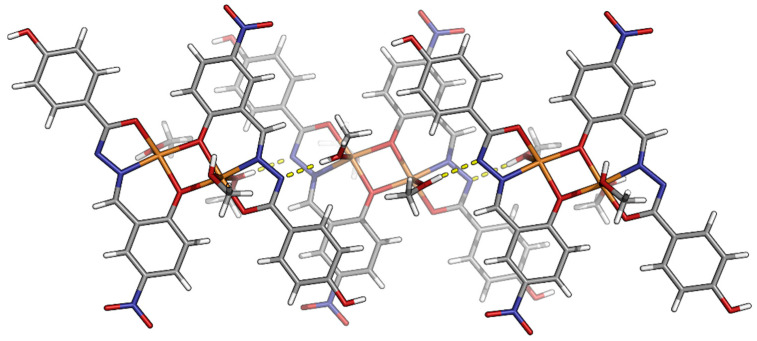
The chains of dinuclear complexes are connected via hydrogen bonds of *R*^2^_2_(8) type. Hydrogen bonds are represented as an array of yellow cylinders.

**Figure 4:**
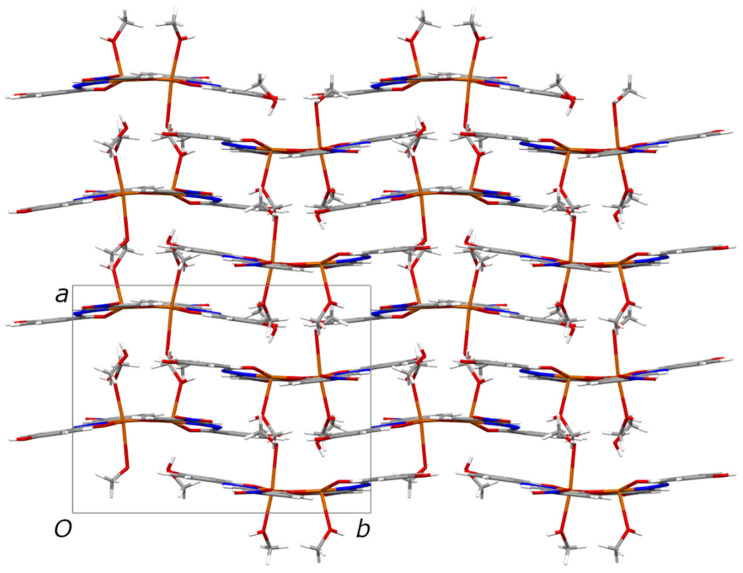
The crystal packing viewed along the crystallographic *c* axis.

The crystal structure also includes additional methanol molecules as crystallization solvents. These molecules engage in hydrogen bonding, acting as donors to the O12 atom and acceptors to the hydroxyl group O13 of neighbouring molecules (Table 3, Figure 3 and Figure 4). Notably, the molecular arrangement positions the Cu1 ion in proximity to the O23 atom of an adjacent molecule, with a contact distance of 3.117(2) Å.

The overall crystal structure is intricate, characterized by a network of hydrogen bonds. Among these, *R*^2^_2_(8)-type interactions link the coordination complexes into chains aligned parallel to the crystallographic *b*-axis. Propagation along the other two axes is facilitated by O–H···O hydrogen bonds, formed between methanol molecules and hydroxyl groups. This extensive hydrogen-bonding network contributes to the stability and structural organization of the complex.

### 2.3. Optical Properties

**Figure 5:**
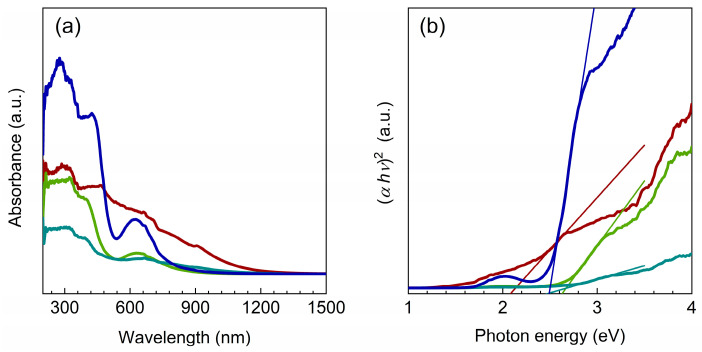
(**a**) Kubelka–Munk diffuse reflectance absorption spectrum of compounds [Cu_2_(L^1^)_2_] (lemon green curve), [Cu_2_(L^2^)_2_(MeOH)_3_] (blue curve), [Cu_2_(L^3^)(H_2_O)_2_] (dark green curve), and [Cu_2_(L^4^)(H_2_O)_2_] (red curve) and (**b**) direct optical transitions. Full lines indicate the band gap energies *E*_g_([Cu_2_(L^1^)_2_]) = 2.68 eV, *E*_g_([Cu_2_(L^2^)_2_]∙3MeOH) = 2.51 eV, *E*_g_([Cu_2_(L^3^)(H_2_O)_2_]) = 2.70 eV, and *E*_g_([Cu_2_(L^4^)(H_2_O)_2_]) = 2.13 eV.

Copper complexes in solid-state [Cu_2_(L^1^)_2_], [Cu_2_(L^2^)_2_(MeOH)_3_], [Cu_2_(L^3^)(H_2_O)_2_], and [Cu_2_(L^4^)(H_2_O)_2_] were analyzed using diffuse reflectance spectroscopy (DRS) at room temperature to examine possible optical transitions; see Figure 5. In the UV region, absorption bands were observed between 280 and 300 nm, attributed to *π* → π* intra-ligand charge transfer [31]. A prominent band at 420 nm corresponds to ligand-to-metal charge transfer (LMCT) [32]. Additionally, a broad intensive peak at 630 nm, especially pronounced for [Cu_2_(L^2^)_2_(MeOH)_3_], can be assigned to the ^2^E_g_ → ^2^T_2g_ transition in the distorted octahedral geometry around Cu(II) ions [33], as discussed in the above subchapter. The optical band gap was estimated for indirect transitions by locating the intersection of the photon energy axis with the extrapolated linear region of the Kubelka–Munk function plot against energy. The resulting values, ranging from 2.13 to 2.70 eV, are in good agreement with the literature reports for copper(II) complexes [34,35]. These results indicate the semiconductor nature of the synthesized complexes, which agrees with their measured electrical properties.

### 2.4. Electrical Properties

This study offers a thorough evaluation of copper-based coordination complexes, specifically [Cu_2_(L^1^)_2_], [Cu_2_(L^2^)_2_(MeOH)_3_], [Cu_2_(L^3^)(H_2_O)_2_], and [Cu_2_(L^4^)(H_2_O)_2_], with the aim of elucidating the factors influencing their behaviour. Key factors such as solvent loss during thermal treatment and the steric properties of the ligands are analyzed to elucidate their contributions to the electrical conductivity of these materials.

Solid-state impedance spectroscopy (IS) [36,37,38] was used to study the electrical behaviour of previously mentioned Cu-based complexes over a broad frequency and temperature range. IS measurements for each complex incorporated a thermal cycling process, given their demonstrated thermal stability and potential decomposition at higher temperatures. Moreover, in correlation to TGA results, the temperature range IS is tuned to cover possible structural transformations (from RT up to a maximal temperature of 230 °C, depending on each analyzed complex). Based on the temperature-dependent conductivity profiles, it is observed that each complex exhibited semiconductor-like behaviour, with conductivity increasing as a function of temperature, while in the following sections, the impact of thermal treatment on the complexes’ electrical behaviour will be discussed.

**Figure 6:**
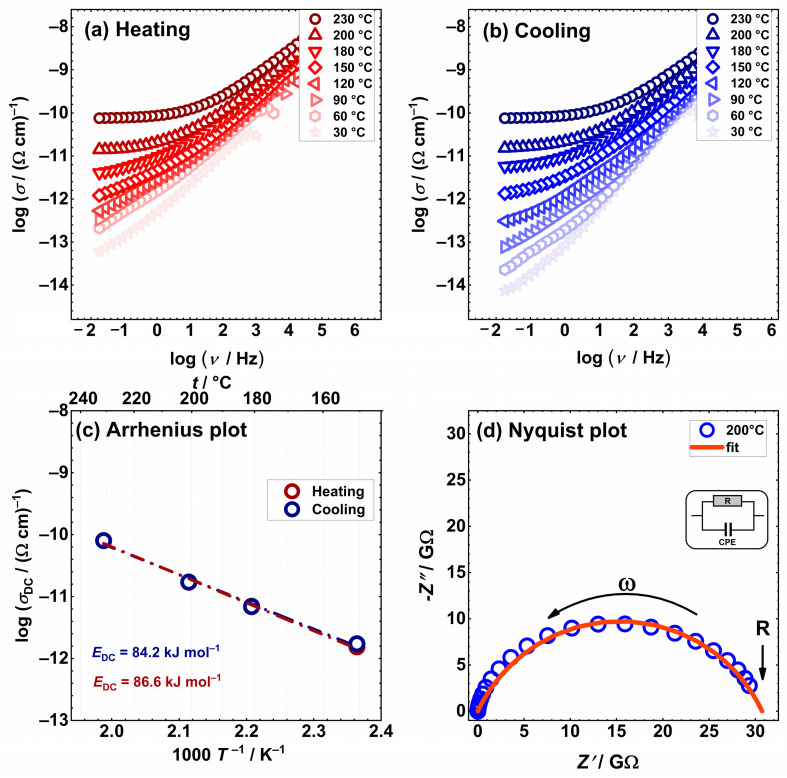
Conductivity spectra for dinuclear [Cu_2_(L^1^)_2_] complex in heating (**a**) and cooling (**b**) cycles; (**c**) Arrhenius plot—temperature dependence of DC conductivity (log(*σ*_DC_) vs. 1000/*T*) for both cycles (red circle—heating; blue circle—cooling); and (**d**) Nyquist plot at 200 °C. The corresponding equivalent circuit in (**d**) used for fitting the data is shown in the inset, and open squares denote experimental values, while a solid orange line corresponds to the best fit. Dash-dot lines in (**c**) represent the least-square linear fits to experimental data.

Figure 6a,b present the conductivity spectra of dinuclear [Cu_2_(L^1^)_2_] complex during both heating and cooling cycles as typical spectra for all studied Cu complexes. As anticipated, the electrical conductivity increases with temperature, which implies semiconductive behaviour with Arrhenius temperature dependence and characteristic activation energy; see Figure 6c. The frequency-independent region present in the spectra at elevated temperatures and lower frequencies is known as the DC conductivity plateau. Its prominence and extent vary across the complexes, reflecting the complexes’ distinct electrical properties. In the high-frequency region, conductivity transitions to a frequency-dependent behaviour, referred to as the dis-persion region (σAC). This transition moves to higher frequencies with a temperature increase (its extent is the DC plateau region) underlying dominant electronic transport. At lower temperatures, due to the limited conductivity of the complexes, the DC conductivity value cannot be directly extracted from conductivity spectra. Instead, in such cases, the value is determined by fitting experimental complex impedance spectra by applying an equivalent circuit (EC) modelling approach and the complex nonlinear least-squares (CNLLS) method.

The so-called Nyquist plot for dinuclear complex [Cu_2_(L^1^)_2_] (the imaginary part plotted as a function of the real part of impedance, *Z*′′ vs. *Z*′), Figure 6d, is analyzed using an appropriate equivalent circuit model, based on a parallel R−CPE circuit, where *R* represents the sample resistance, while *CPE* is a constant phase element that approximates the sample capacitance. The CPE is used instead of a capacitor due to the depressed impedance semicircle that intersects the *x*-axis at point *R*. For example, at 200 °C, *R* equals 3.1 × 10^10^ Ω for [Cu_2_(L^1^)_2_], from which, according to the formula *σ*_*DC*_ = (*d*/*S*)∙(1/*R*), the conductivity can be calculated, being equal to 7.6 × 10^−11^ (Ω cm)^−1^, which agrees well with the value determined directly from the conductivity spectra. This approach ensures accurate extraction of DC conductivity values, particularly where low-temperature conductivity falls below the detectable range of direct graphical analysis.

Moreover, as the studied complexes exhibit temperature dependence of conductivity, it is possible to determine the activation energy from the dependence of DC conductivity, *σ*_DC_, on 1000/*T* and the slope of the line according to the Arrhenius equation:

*σ*_DC_ = *σ*_0_exp(−*E*_DC_/*k*_B_*T*), (1)

where *σ*_DC_ is the DC conductivity, *σ*_0_ is the pre-exponential factor, *E*_DC_ is the activation energy, *k*_B_ is the Boltzmann constant, and *T* is the temperature (K); Figure 6c. Corresponding IS measurements are also performed for all studied complexes. DC conductivity @200 °C and the activation energy values for all studied complexes are given in Table 4. The conductivity values, ranging from 1.7 × 10^−14^ to 3.6 × 10^−9^ (Ω cm)^−1^, align well with our previous studies involving similar ligands but with different metal centres, such as molybdenum and vanadium [18,19,20,21]. Moreover, the activation energy (*E*_DC_) values fall between 68.5 and 84.2 kJ mol^−1^ in the cooling run, which are like those observed in our earlier research [18,19,20,21], and additionally align well with the range of values obtained in studies on various semiconductive materials [39,40,41,42,43,44,45] with dominant electronic conduction mechanism.

**Figure 7:**
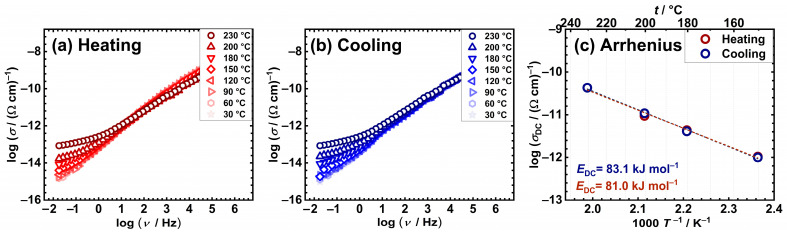
Conductivity spectra for dinuclear [Cu_2_(L^2^)_2_(MeOH)_3_] complex in heating (**a**) and cooling (**b**) cycles, and (**c**) Arrhenius plot—temperature dependence of DC conductivity (log(*σ*_DC_) vs. 1000/*T*) for both cycles (red circle—heating; blue circle—cooling). Dash lines in (**c**) represent the least-square linear fits to experimental data.

The conductivity spectra for dinuclear complex [Cu_2_(L^2^)_2_(MeOH)_3_], involving both heating and cooling runs, along with Arrhenius trends are presented in Figure 7.

In the Cu-based complexes, [Cu_2_(L^1^)_2_] and [Cu_2_(L^2^)_2_(MeOH)_3_], the differences between the heating and cooling cycles are minimal to nearly non-existent, indicating no significant changes in these samples within the studied temperature range. Although [Cu_2_(L^2^)_2_(MeOH)_3_] contains coordinated MeOH that are lost upon heating, the impact is negligible and not visible in the higher temperature range. Based on the TGA data for these two complexes, [Cu_2_(L^1^)_2_] is stable up to 307 °C without any changes after which it decomposes, whereas, in the case of [Cu_2_(L^2^)_2_(MeOH)_3_], one can observe its stability up to 324 °C. However, the signature on the solvent (MeOH) exit in the range from 92 to 118 °C is present in TGA, but in this case, due to low conductivity that is almost temperature-independent below 120 °C, this effect is not visible in the IS data.

**Figure 8:**
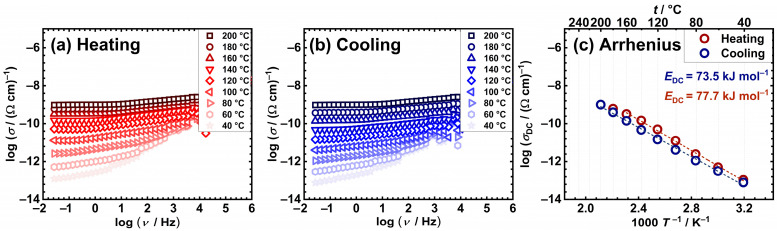
Conductivity spectra for [Cu_2_(L^3^)(H_2_O)_2_] complex in heating (**a**) and cooling (**b**) cycles, and (**c**) Arrhenius plot—temperature dependence of DC conductivity (log(*σ*_DC_) vs. 1000/*T*) for both cycles (red circle—heating; blue circle—cooling). Dash lines in (**c**) represent the least-square linear fits to experimental data.

**Figure 9:**
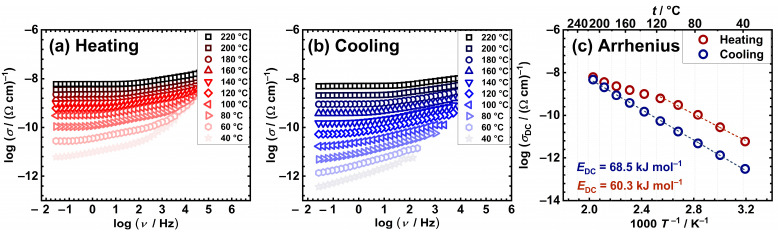
Conductivity spectra for [Cu_2_(L^4^)(H_2_O)_2_] complex in heating (**a**) and cooling (**b**) cycles, and (**c**) Arrhenius plot—temperature dependence of DC conductivity (log(*σ*_DC_) vs. 1000/*T*) for both cycles (red circle—heating; blue circle—cooling). Dash lines in (**c**) represent the least-square linear fits to experimental data.

Going further with a detailed IS study, compounds [Cu_2_(L^3^)(H_2_O)_2_] and [Cu_2_(L^4^)(H_2_O)_2_] exhibit discrepancies during measuring cycles. The representative spectra of the heating vs. cooling run for these two compounds are presented in Figure 8 and Figure 9. Compound [Cu_2_(L^3^)(H_2_O)_2_] shows only slight differences between the heating and cooling cycles, although these are somewhat more pronounced compared to the previously mentioned complexes [Cu_2_(L^1^)_2_] and [Cu_2_(L^2^)_2_(MeOH)_3_]. On the other hand, [Cu_2_(L^4^)(H_2_O)_2_] exhibits more noticeable differences. In both cases, changes in the trend in DC conductivity are observed at temperatures above 120 °C during the heating cycle, likely due to the loss of coordinated water molecules.

Before further comparing the studied complexes, it is important to highlight some key differences among them. The ligands H_2_L^1^ and H_2_L^2^, the so-called ONO donor ligands, are very similar with the only difference being the position of the hydroxyl group. Next is the H_4_L^3^ ligand, also an ONO-NNO donor ligand, and finally the H_4_L^4^ ligand, an ONS-NNO donor ligand with methoxy group on the aldehyde part. While all ligands contribute similarly to the electrical conductivity of the Cu-based complexes, the positions of the functional groups and chains play a central role in the observed properties of electrical transport. All studied complexes are stable across a broad temperature range, consistently maintaining their structure. Notably, complexes [Cu_2_(L^3^)(H_2_O)_2_] and [Cu_2_(L^4^)(H_2_O)_2_], with coordinated water molecules, retain their dinuclear structure. This is in contrast to our previous studies [18,19,20,21], where the loss of coordinated solvent caused structural change that resulted in transformation from a monomeric to a polymeric form. Based on the results from this study on Cu-based complexes, such an exit of solvent does not have a meaningful influence on the electrical transport except for [Cu_2_(L^4^)(H_2_O)_2_], probably due to the S donor atom in the ligand.

The complex [Cu_2_(L^1^)_2_] with the H_2_L^1^ ligand exhibits much higher DC conductivity values compared to the sample with the H_2_L^2^ ligand, [Cu_2_(L^2^)_2_(MeOH)_3_] complex, 7.69 × 10^−11^ vs. 1.72 × 10^−14^ (Ω cm)^−1^, respectively. The difference is likely due to the position of the OH group in the ligand and steric effects. In the H_2_L^1^ ligand, the OH group is in the *ortho* position, which seems to have a positive effect on electron delocalization in the structure compared to when the OH group is in the *para* position, as in H_2_L^2^. Furthermore, in both cases, complexes [Cu_2_(L^1^)_2_] and [Cu_2_(L^2^)_2_(MeOH)_3_] remain dimers, and *E*_DC_ values are almost the same, while the presence/exit of crystalline MeOH does not play an evident role in electrical transport.

Due to the ONO donor similarity, it is fair to discuss the next [Cu_2_(L^3^)(H_2_O)_2_] complex. Its conductivity values are close to the [Cu_2_(L^1^)_2_] complex (10^−10^ vs. 10^−11^(Ω cm)^−1^, respectively); however, its *E*_DC_ values are notably lower (73.5 kJ mol^−1^ vs. 84.2 and 83.1 kJ mol^−1^ [Cu_2_(L^1^)_2_] and [Cu_2_(L^2^)_2_(MeOH)_3_], respectively). The similarity in conductivity values may stem from the binding mode of the ligand to the metal centre, as well as the position of the OH group, again in the *ortho* position. Moreover, it is known that the presence of coordinated water significantly impacts electron transport through the structure, which could account for the lower activation energy observed for the [Cu_2_(L^3^)(H_2_O)_2_] complex compared to the [Cu_2_(L^1^)_2_] complex [18,19,20,21].

Finally, the compound [Cu_2_(L^4^)(H_2_O)_2_] with a ONS-NNO donor ligand exhibits among all studied complexes the highest DC conductivity value and the lowest *E*_DC_ values; see Table 4. There are several properties of sulphur in comparison to oxygen that could be the reason for better charge transfer through complex [Cu_2_(L^4^)(H_2_O)_2_]. Due to its lower electronegativity and larger atomic size, sulphur is a more effective electron donor than oxygen. This facilitates electron transfer and improves charge mobility, ultimately enhancing the conductivity of complexes, especially those involving sulphide or thiol groups, compared to oxygen-containing counterparts [46]. Furthermore, unlike other studied Cu-complexes, this one exhibits the largest disparity between heating and cooling cycles. Although smaller than in previous studies [18,19,20,21], lower conductivity during cooling is observed along with a corresponding increase in activation energy (60.3 vs. 68.5 kJ mol^−1^ in the cooling cycle).

Here, it is important to highlight the correlation between the electrical properties of the copper-based complexes in this study and those of the analogous molybdenum complexes with the same H_2_L^1^ and H_2_L^2^ ligands, as reported in Sarjanović et al. [18]. Firstly, in [18], three out of four molybdenum complexes were monomers with coordinated solvent molecules (MeOH or H_2_O), which leave the coordination sphere of molybdenum during the heating cycle, resulting in the formation of polymers: Activation energy values were approximately 20 kJ mol^−1^ lower than those of the Cu analogues, but the conductivity values are within a similar range (1.82 × 10^−9^ vs. 7.6 × 10^−11^, and 1.52 × 10^−14^ vs. 1.7 × 10^−14^ (Ω cm)^−1^ for the H_2_L^1^ and H_2_L^2^ complexes, respectively). Additionally, similar structural changes from monomer to polymer, as well as comparable conductivity and *E*_DC_ values, were observed in molybdenum complexes with a ligand closely related to H_2_L^1or2^ (lacking the OH group at the R position) reported by Pisk et al. [19]. This result and observation demonstrate that the same ligand, when coordinated to different metal centres and forming distinct initial complex structures, underlines the significance of structural (in)stability with temperature and its resulting impact on electrical properties.

## 3. Materials and Methods

### 3.1. Preparation

The starting compounds used commercially available aldehydes (2-hydroxy-5-nitrobenzaldehyde, 2-2ydroxybenzaldehyde, 2-hydroxy-4-methoxybenzaldehyde) and hydrazides (2-hydroxybenzhydrazide, 4-hydroxybenzhydrazide,carbohydrazide and thiocarbohydrazide), copper(II) acetate monohydrate (Aldrich, St. Louis, MO, USA), and the solvent MeOH (Aldrich, St. Louis, MO, USA), without any purification. The ligands were prepared and characterized according to the published procedures [18,22,23,24,25].

#### Preparation of the Complexes

**Synthesis of dinuclear complexes [Cu_2_(L^1^)_2_] and [Cu_2_(L^2^)_2_(MeOH)_3_]**

A 0.05 g quantity of ligand H_2_L^1or2^ was dissolved in 40 mL of methanol and 0.0349 g of [Cu(OAc)_2_]∙H_2_O was added with reflux for three hours. The precipitate was formed during the reaction and was filtered.

**Complex [Cu_2_(L^1^)_2_]:** green powder, yield (24.5%).

IR-ATR bands *ν*/cm^−1^: 1567 (−C=N_imine_), 1236 (−C−O_phenol_), 664 (Cu−O)

EA for C_28_H_18_Cu_2_N_6_O_10_: C_theo_: 46.35, C_found_: 45.30, H_theo_: 2.50, H_found_: 2.31, N_theo_: 11.58, N_found_: 10.92%

TG: *w*_t_ (CuO, [Cu_2_(L^1^)_2_]) = 21.87%, *w*_eksp_. (CuO, [Cu_2_(L^1^)_2_]) = 19.25%.

The same complex can be obtained if methanol is substituted by acetonitrile.

**Complex [Cu_2_(L^2^)_2_(MeOH)_3_]:** intense green powder, yield (34%).

IR-ATR bands *ν*/cm^−1^: 1602 (−C=N_imine_), 1271 (−C−O_phenol_), 640 (Cu−O),

EA for C_31_H_30_Cu_2_N_6_O_13_: C_theo_: 45.31, C_found_: 44.19, H_theo_: 3.68, H_found_: 2.87, N_theo_: 10.23, N_found_: 9.82%

TG: *w*_t_ (MeOH, [Cu_2_(L^2^)_2_(MeOH)_3_]) = 11.72%, *w*_eksp._ (MeOH, [Cu_2_(L^2^)_2_(MeOH)_3_] = 12.87%, *w*_t_ (CuO, [Cu_2_(L^2^)_2_(MeOH)_3_] = 19.40%, *w*_eksp._ (CuO, [Cu_2_(L^2^)_2_(MeOH)_3_]): = 18.30%

**Synthesis of dinuclear complexes [Cu_2_(L^3or4^)(H_2_O)_2_]**

A 0.095 g (0.25 mmol) quantity of the H_4_L^3or4^ ligand was placed in 50 mL methanol. Then, 0.102 g (0.50 mmol) of [Cu(OAc)_2_]∙H_2_O was added to the suspension. The green suspension was mixed with a stirrer and refluxed for 2.5 h. The resulting olive-green precipitate was filtered and air-dried to a constant mass.

**Complex [Cu_2_(L^3^)(H_2_O)_2_]:** green powder, yield (63.1%).

IR-ATR bands *ν*/cm^−1^: 3308 (O−H); 2941, 1447 (C−H), 1601 (−C=N_imine_); 1244 (−C−O_phenol_), 752, 421(Cu−O)

EA for C_15_H_14_Cu_2_N_4_O_5_: C_theo_: 39.39, C_found_: 39.11, H_theo_: 3.09, H_found_: 2.87, N_theo_: 12.25, N_found_: 11.68%

TG: *w*_t_ (H_2_O, [Cu_2_(L)(H_2_O)_2_]) = 7.89%, *w*_eksp_ (H_2_O, [Cu_2_(L)(H_2_O)_2_]) = 6.4%, *w*_t_ (CuO, [Cu_2_(L)(H_2_O)_2_]) = 34.80% *w*_eksp_(Cu, [Cu_2_(L)(H_2_O)_2_]) = 33.0%

**Complex [Cu_2_(L^4^)(H_2_O)_2_]:** green powder, yield (69.2%).

IR-ATR bands *ν*/cm^−1^: 3308 (O−H); 2941, 1447 (C−H), 1597 (−C=N_imine_); 1242 (−C−O_phenol_), 730, 427(Cu−O)

EA for C_17_H_18_Cu_2_N_4_O_6_S: C_theo_: 38.27, C_found_: 37.74, H_theo_: 3.40, H_found_: 2.76, N_theo_: 10.50, N_found_: 10.10%

TG: *w*_t_ (H_2_O, [Cu_2_(L)(H_2_O)_2_]) = 6.76%, *w*_eksp_ (H_2_O, [Cu_2_(L)(H_2_O)_2_]) = 5.97%, *w*_t_ (CuO, [Cu_2_(L^4^)(H_2_O)_2_]) = 29.90%, *w*_eksp_(Cu, [Cu_2_(L^4^)(H_2_O)_2_]) = 31.5%

### 3.2. Impedance Spectroscopy Measurements

The electrical and dielectric properties of the compounds [Cu_2_(L^1^)_2_], [Cu_2_(L^2^)_2_]∙3MeOH, [Cu_2_(L^3^)(H_2_O)_2_], and [Cu_2_(L^4^)(H_2_O)_2_] were studied via impedance spectroscopy (IS). Complex impedance was measured over a wide range of frequencies (0.01 Hz to 1 MHz) and temperatures (30–230 °C) using an impedance analyzer (Novocontrol Alpha-AN Dielectric Spectrometer, Novocontrol Technologies GmbH & Co. KG, Hundsangen, Germany). The temperature was controlled to ±0.2 °C. The measurements were performed on polycrystalline powder samples pressed into cylindrical pellets with a diameter of 5 mm diameter and a thickness of ~1 mm under a uniform load using a hydraulic press. For the electrical contact, gold electrodes were sputtered onto both sides of the pellets using an SC7620 sputter coater from Quorum Technologies (Laughton, UK). The experimental data were analyzed by electrical equivalent circuit (EEC) modelling using the complex nonlinear least-squares (CNLLSQ) fitting procedure using the WinFIT software [47].

### 3.3. Physical Methods

Elemental analyses were conducted by the Analytical Services Laboratory of the Ruđer Bošković Institute, Zagreb.

Thermogravimetric (TG) analyses were performed using a Mettler TGA/DSC3+ thermobalance in Al_2_O_3_ crucibles. All experiments were performed in an oxygen atmosphere with a flow rate of 200 cm^3^ min^−1^ and with heating rates of 10 K min^−1^ and analyzed with the Mettler STARe 9.01 software.

IR-ATR spectra were recorded on a Perkin Elmer Spectrum Two spectrometer (Perkin Elmer, Waltham, MA, USA) in the spectral range between 4500 and 450 cm^−1^.

UV-Vis diffuse reflectance spectra were recorded at 293 K using a UV-Vis-NIR spectrometer (model UV-3600, Shimadzu, Japan) equipped with an integrated sphere. Barium sulphate was used as a reference. The diffuse reflectance spectra were transformed using the Kubelka–Munk function. Tauc plots were used to calculate the optical band gap energy.

Single crystals of [Cu_2_(L^2^)_2_(MeOH)_3_]∙MeOH of an appropriate quality were selected for the diffraction experiments. SCXRD experiments were performed using a Rigaku XtaLAB Synergy-S diffractometer (Rigaku, Tokyo, Japan) using Cu Kα radiation, *λ* = 1.54184 Å, and a HyPix detector. Diffracted intensities were collected at 170 K, and data were processed using the CrysAlisPro v171.42.49 program package [48]. A summary of the general crystallographic data is presented in Table 1. The structures were solved using SHELXT [49]. The refinement was performed via full-matrix least-squares methods based on *F*^2^ values against all reflections, including the anisotropic displacement parameters for all non-H atoms. Hydrogen atoms attached to carbon atoms were placed in geometrically idealized positions and refined by using the riding model, with *U*_iso_ = 1.2 *U*_eq_ of the connected carbon atom, or as ideal CH_3_ groups, with *U*_iso_ = 1.5 *U*_eq_. All refinements were conducted using SHELXL [50]. The SHELX programs were operated within the Olex2 suit [51]. Geometrical calculations were performed by Platon [52], and molecular graphics were produced using the Mercury 2021.3.0 software [53]. CCDC 2411599 contains the supplementary crystallographic data for this paper. These data can be obtained free of charge from The Cambridge Crystallographic Data Centre via www.ccdc.cam.ac.uk/structures (accessed on 19 December 2024).

## 4. Conclusions

The presented research reports the preparation and characterization of novel Cu-based complexes, [Cu_2_(L^1^)_2_], [Cu_2_(L^2^)_2_(MeOH)_3_]MeOH, [Cu_2_(L^3^)(H_2_O)_2_], and [Cu_2_(L^4^)(H_2_O)_2_]. The ligands selected for this study were strategically chosen to examine two critical factors: (1) the effect of hydroxyl group positioning on the aroyl ring of hydrazone (H_2_L^1^ vs. H_2_L^2^) and (2) the influence of carbonyl versus thiocarbonyl groups in the hydrazide part of ligands, alongside variations in the substituents on the aldehyde moiety of hydrazone (H_4_L^3^ vs. H_4_L^4^).

The study highlights the influence of ligand structure and metal coordination on the electrical properties of copper-based complexes. Complex [Cu_2_(L^1^)_2_] exhibits significantly higher DC conductivity (7.69 × 10^−11^ Ω^−1^ cm^−1^) compared to [Cu_2_(L^2^)_2_(MeOH)_3_] (1.72 × 10^−14^ Ω^−1^ cm^−1^), likely due to the ortho position of the OH group enhancing electron delocalization. Complex [Cu_2_(L^3^)(H_2_O)_2_] shows similar conductivity to [Cu_2_(L^1^)_2_] (10^−10^ vs. 10^−11^ Ω^−1^ cm^−1^, respectively) but has a lower activation energy (73.5 kJ mol^−1^ vs. 84.2 kJ mol^−1^). The ONS-NNO donor ligand in [Cu_2_(L^4^)(H_2_O)_2_] achieves the highest DC conductivity (~10^−8^ Ω^−1^ cm^−1^) and the lowest activation energy (60.3 kJ mol^−1^ during heating), attributed to sulphur’s superior electron-donating properties. Comparisons with previously studied molybdenum complexes reveal similar conductivity ranges, with molybdenum showing lower activation energies (~20 kJ mol^−1^). These findings underscore the significant interplay between ligand positioning, metal centres, and structural stability on electrical properties. 

## Figures and Tables

**Table 1:** Crystal data and structure refinement for [Cu_2_(L^2^)_2_(MeOH)_3_]∙MeOH.

Empirical formula	C_32_H_34_Cu_2_N_6_O_14_
Formula weight	853.73
Temperature/K	169.99 (10)
Crystal system	monoclinic
Space group	*P*2_1_/*n*
*a*/Å	13.40491 (17)
*b*/Å	17.4015 (2)
*c*/Å	14.6893 (2)
*α*/°	90
*β*/°	97.3201 (13)
*γ*/°	90
*V*/Å^3^	3398.58 (8)
*Z*	4
*ρ*_calc_ g/cm^3^	1669
*μ*/mm^−1^	2252
*F*(000)	1752.0
Crystal size/mm^3^	0.157 × 0.045 × 0.025
Radiation	Cu Kα (*λ* = 1.54184)
2*Θ* range for data collection/°	7.914 to 159.922
Index ranges	−16 ≤ *h* ≤ 17, −20 ≤ *k* ≤ 22, −18 ≤ *l* ≤ 18
Reflections collected	31,245
Independent reflections	7256 [*R*_int_ = 0.0699, *R*_sigma_ = 0.0525]
Data/restraints/parameters	7256/3/499
Goodness of fit on *F*^2^	1042
Final *R* indexes [*I* ≥ 2*σ*(*I*)]	*R*_1_ = 0.0487, *wR*_2_ = 0.1178
Final *R* indexes [all data]	*R*_1_ = 0.0653, *wR*_2_ = 0.1252
Largest diff. peak/hole/e Å^−3^	0.64/−0.70
CCDC deposition number	2,411,599

**Table 2:** Bond lengths for [Cu_2_(L^2^)_2_(MeOH)_3_]∙MeOH.

Atom1	Atom2	Length/Å
Cu1	O1M	2.352 (2)
Cu1	O11	1.9491 (18)
Cu1	O12	1.9416 (17)
Cu1	O21	1.9680 (17)
Cu1	N11	1.916 (2)
Cu2	O2M	2.419 (2)
Cu2	O11	1.9990 (17)
Cu2	O21	1.9461 (18)
Cu2	O22	1.9133 (19)
Cu2	N21	1.932 (2)
Cu2	O4M	2.733 (2)

**Table 3:** Geometry of hydrogen bonds (Å, °) for [Cu_2_(L^2^)_2_(MeOH)_3_]∙MeOH.

D–H···A	D–H	H···A	D···A	∠D–H···A	Symmetry Code
O1M–H1M···N12	0.87 (2)	1.98 (2)	2.840 (3)	173 (2)	1 − *x*, 1 − *y*, 1 − *z*
O2M–H2M···N22	0.8400	2.0300	2.864 (3)	177	1 − *x*, 2 − *y*, 1 − *z*
O3M–H3M···O12	0.8400	1.9900	2.819 (3)	170	3/2 − *x*, −1/2 + *y*, 1/2 − *z*
O4M–H4M···O13	0.8400	1.9400	2.765 (3)	169	3/2 − *x*, 1/2 + *y*, 1/2 − *z*
O13–H13···O3M	0.8400	1.8100	2.649 (3)	176	-
O23–H23···O4M	0.8400	1.8700	2.676 (3)	160	3/2 − *x*, 1/2 + *y*, 3/2 − *z*

**Table 4:** Electrical parameters of the obtained Cu-based complexes.

Sample	^a^ *σ*_DC_/(Ω cm)^−1^	*E*_DC_/kJ mol^−1^ (Heating Run)	*E*_DC_/kJ mol^−1^ (Cooling Run)
[Cu_2_(L^1^)_2_]	7.6 × 10^−11^	86.6	84.2
[Cu_2_(L^2^)_2_(MeOH)_3_]	1.7 × 10^−14^	81.0	83.1
[Cu_2_(L^3^)(H_2_O)_2_]	6.1 × 10^−10^	77.7	73.5
[Cu_2_(L^4^)(H_2_O)_2_]	3.6 × 10^−9^	60.3	68.5

^a^ measured @200 °C.

## Data Availability

The data presented in this study are available from the corresponding author upon request.
